# Supplementary material for: Schizophrenia risk and reproductive success: a Mendelian randomization study
Source: R Soc Open Sci. 2019 Mar 6;6(3):181049. doi: 10.1098/rsos.181049 (PMC6458425; doi:10.1098/rsos.181049)
Supplement: Supplementary material [file rsos181049supp1.doc]

**Supplementary material**

**Supplementary Text:**

For genetic liability for schizophrenia, 75 of the 128 SNPs were not available in our outcome dataset UK Biobank. We found proxies for 48 SNPs, using a linkage disequilibrium r^2^ of 0.8 or above, through SNIPA (http://snipa.helmholtz-muenchen.de/snipa3/) or proxies used previously (1). Where palindromic SNPs were used, the minor allele frequency (MAF) was checked to ensure there were no issues with strand mismatches. There were no palindromic SNPs with MAF around 0.5. We used SNIPA (http://snipa.helmholtz-muenchen.de/snipa3/) with a linkage disequilibrium (LD) threshold of 0.2 to check SNP independence. We natural log transformed all schizophrenia OR’s prior to analysis.

For educational attainment analysis, 69 independent SNPs were available in UK Biobank data. Two palindromic SNPs with MAF close to 0.5 were excluded due to strand ambiguities. We therefore used 67 SNPs associated with educational attainment.

Cochran’s Q for an IVW approach of educational attainment and number of children was 199.54, and 144.88 for age at first birth, suggesting overdispersion although this appeared balanced. Cochran’s Q was 156.48 and 286.64 and 301.88 for genetic liability of schizophrenia and number of children, age at first birth and number of sexual partner analysis, respectively, suggesting overdispersion although this again appeared balanced.

**Table S1.** List of SNPs associated with schizophrenia (p < 5 × 10^-8^) and associations with number of children, age at first birth and number of sexual partners.

| **SNP** | **Original SNP if proxy used** | **r^2^ for proxy** | **Schizophrenia^a^** | | **Number of offspring^b^** | | **Age at first birth^c^** | | **Number of sexual partners** | |
| --- | --- | --- | --- | --- | --- | --- | --- | --- | --- | --- |
|  |  |  | **Ln(OR)** | **SE** | **β** | **SE** | **β** | **SE** | **β** | **SE** |
| rs1009080 | rs1498232 | 0.99 | -0.071 | 0.012 | 0.004 | 0.003 | -0.018 | 0.020 | -0.021 | 0.024 |
| rs1023500 |  |  | 0.076 | 0.014 | 0.002 | 0.004 | -0.024 | 0.023 | -0.025 | 0.028 |
| rs10412446 | rs56873913 | 0.97 | 0.057 | 0.013 | 0.001 | 0.003 | 0.040 | 0.022 | 0.021 | 0.027 |
| rs10503253 |  |  | 0.072 | 0.013 | 0.004 | 0.004 | 0.008 | 0.023 | -0.030 | 0.028 |
| rs10504857 | rs7819570 | 1.00 | -0.074 | 0.014 | 0.002 | 0.004 | -0.018 | 0.024 | -0.026 | 0.030 |
| rs10520163 |  |  | 0.058 | 0.011 | -0.004 | 0.003 | 0.007 | 0.018 | 0.019 | 0.022 |
| rs10779702 | chr1_8424984_D | 0.97 | 0.063 | 0.011 | 0.002 | 0.003 | -0.049 | 0.019 | -0.021 | 0.023 |
| rs10791097 |  |  | 0.077 | 0.011 | 0.007 | 0.003 | -0.024 | 0.018 | 0.009 | 0.022 |
| rs10803138 |  |  | -0.072 | 0.013 | 0.006 | 0.003 | -0.053 | 0.021 | -0.086 | 0.026 |
| rs10860964 |  |  | 0.063 | 0.011 | 0.005 | 0.003 | -0.035 | 0.019 | 0.026 | 0.023 |
| rs10900851 | rs10043984 | 0.99 | -0.064 | 0.012 | 0.008 | 0.003 | -0.052 | 0.021 | 0.023 | 0.026 |
| rs10933068 | rs11685299 | 1.00 | -0.063 | 0.012 | -0.008 | 0.003 | 0.015 | 0.020 | -0.083 | 0.024 |
| rs11027857 |  |  | 0.064 | 0.011 | 0.005 | 0.003 | 0.007 | 0.018 | -0.002 | 0.022 |
| rs1106568 |  |  | -0.069 | 0.013 | -0.005 | 0.003 | 0.070 | 0.021 | 0.020 | 0.026 |
| rs11139497 |  |  | 0.066 | 0.012 | -0.005 | 0.003 | -0.050 | 0.019 | 0.032 | 0.024 |
| rs11210892 |  |  | -0.068 | 0.012 | -0.001 | 0.003 | 0.084 | 0.019 | -0.083 | 0.024 |
| rs1160682 | rs12129573 | 1.00 | -0.068 | 0.011 | -0.003 | 0.003 | 0.053 | 0.019 | -0.003 | 0.023 |
| rs11632947 | rs12903146 | 0.99 | 0.066 | 0.011 | 0.001 | 0.003 | 0.012 | 0.018 | 0.023 | 0.022 |
| rs11682175 |  |  | -0.073 | 0.011 | 0.002 | 0.003 | -0.076 | 0.018 | 0.043 | 0.022 |
| rs11683083 | chr2_146436222_I | 1.00 | -0.078 | 0.014 | 0.007 | 0.004 | -0.039 | 0.024 | -0.043 | 0.029 |
| rs12063329 | rs140505938 | 1.00 | 0.088 | 0.015 | -0.001 | 0.004 | 0.014 | 0.024 | 0.036 | 0.030 |
| rs12148337 |  |  | 0.057 | 0.011 | 0.002 | 0.003 | 0.001 | 0.018 | -0.028 | 0.022 |
| rs12325245 |  |  | -0.086 | 0.016 | -0.001 | 0.004 | -0.058 | 0.026 | -0.019 | 0.032 |
| rs12421382 |  |  | -0.065 | 0.012 | -0.004 | 0.003 | -0.024 | 0.020 | -0.029 | 0.024 |
| rs12522290 |  |  | 0.082 | 0.015 | 0.008 | 0.004 | -0.025 | 0.024 | 0.020 | 0.029 |
| rs12619354 | rs59979824 | 0.87 | 0.059 | 0.012 | -0.004 | 0.003 | 0.040 | 0.019 | 0.062 | 0.024 |
| rs12654855 | rs79212538 | 0.95 | -0.128 | 0.025 | -0.010 | 0.007 | 0.023 | 0.043 | -0.083 | 0.053 |
| rs12659129 | chr5_140143664_I | 1.00 | 0.052 | 0.011 | -0.002 | 0.003 | -0.019 | 0.018 | -0.037 | 0.022 |
| rs12716972 | rs12691307 | 0.98 | 0.063 | 0.011 | -0.002 | 0.003 | -0.0004 | 0.018 | -0.014 | 0.023 |
| rs13074054 | chr3_180594593_I | 0.99 | 0.077 | 0.014 | 0.008 | 0.004 | -0.008 | 0.022 | -0.041 | 0.027 |
| rs13107325 | rs35518360 | 0.85 | 0.152 | 0.022 | 0.001 | 0.006 | -0.038 | 0.035 | -0.134 | 0.043 |
| rs1501357 |  |  | -0.069 | 0.014 | -0.002 | 0.004 | 0.050 | 0.024 | -0.065 | 0.029 |
| rs16867576 |  |  | 0.096 | 0.017 | -0.009 | 0.004 | -0.028 | 0.027 | 0.149 | 0.033 |
| rs17049247 | rs75575209 | 0.97 | -0.103 | 0.019 | 0.008 | 0.005 | -0.091 | 0.032 | 0.024 | 0.039 |
| rs17149781 | chr7_24747494_D | 0.91 | -0.086 | 0.017 | 0.004 | 0.005 | -0.083 | 0.029 | 0.007 | 0.036 |
| rs17194490 |  |  | 0.097 | 0.015 | -0.003 | 0.004 | 0.034 | 0.025 | 0.048 | 0.030 |
| rs17273111 | rs4330281 | 1.00 | 0.056 | 0.011 | -0.001 | 0.003 | -0.025 | 0.018 | 0.020 | 0.022 |
| rs17594526 | rs78322266 | 1.00 | 0.169 | 0.031 | -0.003 | 0.009 | 0.053 | 0.056 | 0.169 | 0.069 |
| rs17602354 | rs72934570 | 0.92 | 0.141 | 0.021 | -0.001 | 0.005 | 0.010 | 0.033 | 0.188 | 0.040 |
| rs1782810 | rs1702294 | 0.99 | 0.118 | 0.014 | 0.003 | 0.004 | 0.004 | 0.023 | 0.000 | 0.029 |
| rs2007044 |  |  | -0.092 | 0.011 | 0.002 | 0.003 | -0.015 | 0.019 | -0.007 | 0.023 |
| rs2053079 |  |  | -0.072 | 0.013 | 0.004 | 0.003 | -0.037 | 0.021 | 0.058 | 0.026 |
| rs2057070 | rs9607782 | 0.81 | -0.068 | 0.012 | 0.006 | 0.003 | -0.051 | 0.020 | 0.029 | 0.025 |
| rs2068012 |  |  | -0.070 | 0.013 | -0.002 | 0.003 | 0.048 | 0.022 | -0.007 | 0.027 |
| rs211829 |  |  | 0.054 | 0.011 | -0.005 | 0.003 | 0.001 | 0.019 | 0.022 | 0.023 |
| rs215411 |  |  | 0.069 | 0.012 | -0.002 | 0.003 | 0.003 | 0.020 | 0.017 | 0.024 |
| rs2239063 |  |  | 0.069 | 0.012 | -0.0002 | 0.003 | 0.003 | 0.020 | 0.053 | 0.025 |
| rs2296569 | rs55833108 | 0.83 | -0.068 | 0.014 | 0.001 | 0.004 | -0.007 | 0.023 | 0.074 | 0.028 |
| rs2514218 |  |  | -0.072 | 0.012 | -0.0004 | 0.003 | 0.027 | 0.019 | 0.005 | 0.024 |
| rs2535627 |  |  | 0.070 | 0.011 | -0.0004 | 0.003 | -0.019 | 0.018 | -0.019 | 0.023 |
| rs2693698 |  |  | -0.062 | 0.011 | 0.001 | 0.003 | 0.025 | 0.018 | -0.006 | 0.023 |
| rs2796275 | rs7523273 | 0.98 | 0.053 | 0.012 | 0.001 | 0.003 | -0.002 | 0.019 | 0.037 | 0.024 |
| rs2851447 |  |  | -0.084 | 0.012 | -0.002 | 0.003 | -0.035 | 0.021 | -0.048 | 0.026 |
| rs2955357 | rs8082590 | 1.00 | 0.064 | 0.012 | -0.001 | 0.003 | 0.031 | 0.020 | -0.038 | 0.024 |
| rs2965180 | rs2905426 | 0.97 | 0.063 | 0.011 | 0.009 | 0.003 | -0.068 | 0.019 | -0.006 | 0.023 |
| rs2973161 | rs2973155 | 0.97 | -0.069 | 0.011 | -0.006 | 0.003 | 0.006 | 0.019 | -0.033 | 0.023 |
| rs324015 | rs324017 | 0.81 | -0.069 | 0.013 | 0.003 | 0.003 | 0.005 | 0.021 | -0.016 | 0.026 |
| rs3802924 | rs75059851 | 0.95 | 0.088 | 0.014 | -0.0003 | 0.004 | -0.041 | 0.023 | 0.005 | 0.029 |
| rs3849046 |  |  | 0.062 | 0.011 | -0.008 | 0.003 | 0.010 | 0.019 | 0.005 | 0.023 |
| rs4128242 | chr18_52749216_D | 1.00 | 0.067 | 0.011 | 0.003 | 0.003 | -0.024 | 0.019 | 0.021 | 0.023 |
| rs4129585 |  |  | 0.079 | 0.011 | 0.003 | 0.003 | -0.035 | 0.018 | 0.036 | 0.022 |
| rs4240748 |  |  | -0.057 | 0.011 | 0.009 | 0.003 | 0.030 | 0.019 | -0.027 | 0.023 |
| rs436124 | rs679087 | 1.00 | 0.061 | 0.011 | 0.002 | 0.003 | 0.012 | 0.019 | 0.034 | 0.023 |
| rs4388249 |  |  | 0.067 | 0.014 | 0.001 | 0.004 | -0.037 | 0.025 | 0.104 | 0.031 |
| rs4391122 |  |  | -0.078 | 0.011 | -0.001 | 0.003 | 0.068 | 0.018 | 0.027 | 0.023 |
| rs4518583 | rs3735025 | 1.00 | 0.061 | 0.011 | -0.004 | 0.003 | 0.061 | 0.019 | 0.035 | 0.023 |
| rs4523957 |  |  | 0.070 | 0.012 | -0.005 | 0.003 | -0.007 | 0.019 | 0.055 | 0.024 |
| rs4648845 |  |  | 0.067 | 0.012 | 0.002 | 0.003 | 0.015 | 0.018 | -0.010 | 0.023 |
| rs4664442 | rs2909457 | 0.98 | 0.059 | 0.011 | 0.002 | 0.003 | 0.029 | 0.018 | 0.017 | 0.022 |
| rs4702 |  |  | -0.081 | 0.012 | -0.006 | 0.003 | 0.026 | 0.018 | -0.132 | 0.022 |
| rs6065094 |  |  | -0.075 | 0.012 | -0.003 | 0.003 | -0.008 | 0.019 | -0.062 | 0.024 |
| rs6461049 | chr7_2025096_I | 0.93 | 0.080 | 0.011 | 0.006 | 0.003 | 0.033 | 0.018 | 0.077 | 0.023 |
| rs6466056 | rs6466055 | 1.00 | 0.068 | 0.011 | -0.0005 | 0.003 | -0.052 | 0.019 | 0.062 | 0.023 |
| rs6579959 | rs111294930 | 0.96 | -0.067 | 0.012 | -0.003 | 0.003 | -0.018 | 0.020 | -0.103 | 0.025 |
| rs6670165 |  |  | 0.074 | 0.014 | -0.001 | 0.004 | 0.004 | 0.023 | 0.066 | 0.029 |
| rs6704641 |  |  | 0.075 | 0.015 | 0.006 | 0.004 | 0.008 | 0.023 | -0.071 | 0.029 |
| rs6704768 |  |  | -0.077 | 0.011 | -0.003 | 0.003 | -0.051 | 0.018 | -0.007 | 0.023 |
| rs7085104 | rs11191419 | 0.99 | 0.098 | 0.011 | 0.002 | 0.003 | -0.001 | 0.019 | -0.067 | 0.024 |
| rs7140568 | rs12887734 | 1.00 | 0.085 | 0.012 | 0.002 | 0.003 | -0.004 | 0.020 | 0.036 | 0.025 |
| rs715170 |  |  | -0.067 | 0.012 | -0.001 | 0.003 | 0.025 | 0.021 | -0.061 | 0.025 |
| rs7267348 |  |  | -0.066 | 0.013 | 0.003 | 0.003 | -0.003 | 0.021 | -0.005 | 0.026 |
| rs7432375 |  |  | -0.071 | 0.011 | -0.004 | 0.003 | 0.017 | 0.019 | -0.006 | 0.023 |
| rs7499750 | rs7405404 | 1.00 | 0.077 | 0.013 | -0.005 | 0.003 | 0.018 | 0.022 | 0.069 | 0.027 |
| rs7730110 | rs11740474 | 0.81 | -0.059 | 0.011 | 0.002 | 0.003 | 0.021 | 0.019 | -0.014 | 0.024 |
| rs7801375 |  |  | -0.083 | 0.015 | 0.0003 | 0.004 | 0.018 | 0.025 | 0.009 | 0.031 |
| rs7815859 | rs36068923 | 1.00 | 0.083 | 0.013 | 0.002 | 0.004 | 0.049 | 0.023 | 0.013 | 0.028 |
| rs787983 | rs6434928 | 0.98 | -0.073 | 0.012 | -0.004 | 0.003 | -0.014 | 0.019 | -0.019 | 0.024 |
| rs7893279 |  |  | 0.112 | 0.018 | 0.008 | 0.005 | 0.083 | 0.029 | 0.106 | 0.035 |
| rs7927176 | rs77502336 | 0.96 | -0.059 | 0.012 | 0.00001 | 0.003 | 0.017 | 0.019 | 0.053 | 0.024 |
| rs8042374 |  |  | 0.090 | 0.013 | -0.009 | 0.003 | -0.005 | 0.022 | -0.049 | 0.027 |
| rs8044995 |  |  | 0.077 | 0.014 | 0.002 | 0.004 | -0.022 | 0.025 | 0.017 | 0.030 |
| rs832187 |  |  | -0.070 | 0.011 | 0.0002 | 0.003 | -0.037 | 0.019 | 0.021 | 0.023 |
| rs867743 | rs6984242 | 1.00 | -0.062 | 0.011 | -0.003 | 0.003 | -0.029 | 0.019 | 0.020 | 0.023 |
| rs884808 | rs14403 | 0.86 | -0.054 | 0.013 | 0.003 | 0.004 | 0.040 | 0.022 | -0.058 | 0.027 |
| rs9420 |  |  | 0.058 | 0.011 | -0.002 | 0.003 | -0.007 | 0.019 | 0.024 | 0.024 |
| rs950169 |  |  | -0.079 | 0.012 | -0.003 | 0.003 | 0.038 | 0.021 | -0.023 | 0.025 |
| rs9636107 |  |  | -0.080 | 0.011 | -0.003 | 0.003 | 0.063 | 0.018 | -0.034 | 0.023 |
| rs982256 | rs13240464 | 0.98 | 0.078 | 0.012 | -0.001 | 0.003 | 0.030 | 0.019 | -0.043 | 0.024 |
| rs9841616 |  |  | -0.074 | 0.015 | -0.001 | 0.004 | -0.021 | 0.024 | -0.051 | 0.030 |
| rs9876421 | rs75968099 | 0.93 | 0.079 | 0.011 | -0.007 | 0.003 | 0.021 | 0.019 | 0.043 | 0.024 |
| rs9922678 |  |  | 0.068 | 0.012 | -0.001 | 0.003 | 0.008 | 0.020 | -0.008 | 0.025 |

^a^ Schizophrenia genetic data from the Psychiatric Genomics Consortium GWAS; ^b^ Number of children data from UK Biobank; ^c^ Age at first birth data from UK Biobank; ^d^ Number of sexual partners data from UK Biobank.

**Table S2. List of SNPs associated with educational attainment (p < 5 × 10^-8^).**

| **SNP** | **Educational attainment^a^** | | **Number of offspring^b^** | | **Age at first birth^c^** | |
| --- | --- | --- | --- | --- | --- | --- |
|  | **β** | **SE** | **β** | **SE** | **β** | **SE** |
| rs10061788 | 0.021 | 0.004 | -0.002 | 0.004 | 0.033 | 0.025 |
| rs1008078 | -0.016 | 0.003 | 0.006 | 0.003 | -0.077 | 0.019 |
| rs1043209 | 0.018 | 0.003 | 0.008 | 0.003 | 0.052 | 0.019 |
| rs10496091 | -0.018 | 0.003 | -0.003 | 0.003 | -0.066 | 0.020 |
| rs11191193 | 0.018 | 0.003 | 0.005 | 0.003 | 0.063 | 0.019 |
| rs11210860 | 0.017 | 0.003 | 0.001 | 0.003 | 0.065 | 0.019 |
| rs112634398 | 0.036 | 0.007 | -0.013 | 0.007 | 0.069 | 0.046 |
| rs113520408 | 0.017 | 0.003 | 0.000 | 0.003 | 0.023 | 0.020 |
| rs11588857 | 0.020 | 0.003 | 0.011 | 0.004 | 0.034 | 0.022 |
| rs11689269 | 0.016 | 0.003 | -0.003 | 0.003 | 0.061 | 0.019 |
| rs11690172 | 0.015 | 0.003 | -0.004 | 0.003 | 0.027 | 0.019 |
| rs11712056 | 0.024 | 0.003 | -0.013 | 0.003 | 0.138 | 0.018 |
| rs11768238 | -0.017 | 0.003 | -0.002 | 0.003 | -0.010 | 0.020 |
| rs12531458 | 0.014 | 0.003 | -0.003 | 0.003 | 0.014 | 0.018 |
| rs12646808 | 0.016 | 0.003 | 0.001 | 0.003 | 0.054 | 0.020 |
| rs12671937 | 0.016 | 0.003 | -0.003 | 0.003 | 0.034 | 0.018 |
| rs12772375 | -0.015 | 0.003 | -0.003 | 0.003 | 0.006 | 0.019 |
| rs12969294 | -0.016 | 0.003 | 0.011 | 0.003 | -0.063 | 0.019 |
| rs12987662 | 0.027 | 0.003 | -0.007 | 0.003 | 0.069 | 0.019 |
| rs13294439 | -0.023 | 0.003 | 0.010 | 0.003 | -0.090 | 0.018 |
| rs13402908 | -0.018 | 0.003 | 0.002 | 0.003 | -0.037 | 0.018 |
| rs1402025 | 0.017 | 0.003 | 0.004 | 0.003 | 0.028 | 0.022 |
| rs1606974 | 0.022 | 0.004 | -0.008 | 0.004 | 0.091 | 0.028 |
| rs165633 | -0.018 | 0.003 | 0.000 | 0.004 | 0.004 | 0.022 |
| rs16845580 | 0.016 | 0.003 | -0.006 | 0.003 | 0.039 | 0.019 |
| rs17119973 | -0.019 | 0.003 | 0.005 | 0.003 | -0.054 | 0.021 |
| rs17167170 | 0.020 | 0.003 | -0.005 | 0.004 | 0.073 | 0.023 |
| rs1777827 | 0.015 | 0.003 | -0.004 | 0.003 | 0.032 | 0.019 |
| rs17824247 | -0.016 | 0.003 | 0.007 | 0.003 | -0.057 | 0.019 |
| rs1871109 | -0.016 | 0.003 | 0.000 | 0.003 | -0.019 | 0.018 |
| rs2245901 | -0.016 | 0.003 | 0.000 | 0.003 | -0.052 | 0.019 |
| rs2431108 | 0.016 | 0.003 | 0.006 | 0.003 | 0.024 | 0.019 |
| rs2456973 | -0.020 | 0.003 | 0.010 | 0.003 | -0.096 | 0.019 |
| rs2457660 | -0.017 | 0.003 | 0.002 | 0.003 | -0.061 | 0.019 |
| rs2568955 | -0.017 | 0.003 | 0.009 | 0.004 | -0.022 | 0.024 |
| rs2610986 | -0.016 | 0.003 | -0.007 | 0.003 | -0.014 | 0.020 |
| rs2615691 | -0.037 | 0.007 | 0.019 | 0.008 | 0.095 | 0.050 |
| rs2837992 | 0.015 | 0.003 | -0.006 | 0.003 | 0.050 | 0.019 |
| rs2964197 | 0.015 | 0.003 | -0.007 | 0.003 | 0.032 | 0.018 |
| rs2992632 | 0.017 | 0.003 | -0.005 | 0.003 | 0.047 | 0.020 |
| rs301800 | 0.019 | 0.003 | -0.004 | 0.004 | 0.058 | 0.024 |
| rs3101246 | -0.015 | 0.003 | 0.004 | 0.003 | -0.024 | 0.019 |
| rs324886 | -0.015 | 0.003 | 0.009 | 0.003 | -0.056 | 0.019 |
| rs34072092 | 0.024 | 0.004 | -0.007 | 0.005 | 0.074 | 0.029 |
| rs34305371 | 0.035 | 0.005 | -0.006 | 0.005 | 0.073 | 0.030 |
| rs35761247 | 0.034 | 0.006 | -0.020 | 0.006 | 0.197 | 0.039 |
| rs4493682 | 0.019 | 0.004 | -0.010 | 0.004 | 0.082 | 0.024 |
| rs4500960 | -0.016 | 0.003 | 0.001 | 0.003 | 0.021 | 0.018 |
| rs4851251 | -0.017 | 0.003 | 0.004 | 0.003 | -0.067 | 0.021 |
| rs4863692 | 0.018 | 0.003 | 0.001 | 0.003 | 0.090 | 0.019 |
| rs55830725 | -0.022 | 0.004 | -0.011 | 0.004 | -0.006 | 0.024 |
| rs56231335 | -0.017 | 0.003 | 0.001 | 0.003 | -0.047 | 0.019 |
| rs572016 | 0.014 | 0.003 | 0.005 | 0.003 | 0.042 | 0.018 |
| rs61160187 | -0.017 | 0.003 | -0.002 | 0.003 | -0.060 | 0.019 |
| rs62259535 | 0.048 | 0.008 | -0.011 | 0.008 | 0.251 | 0.049 |
| rs62263923 | -0.016 | 0.003 | 0.021 | 0.003 | -0.052 | 0.019 |
| rs62379838 | 0.016 | 0.003 | -0.004 | 0.003 | -0.007 | 0.020 |
| rs6739979 | -0.015 | 0.003 | 0.001 | 0.003 | -0.048 | 0.019 |
| rs7131944 | 0.015 | 0.003 | -0.001 | 0.003 | -0.015 | 0.019 |
| rs7306755 | 0.023 | 0.003 | 0.004 | 0.004 | 0.039 | 0.023 |
| rs76076331 | 0.020 | 0.004 | -0.001 | 0.004 | 0.092 | 0.028 |
| rs7767938 | 0.017 | 0.003 | 0.000 | 0.003 | 0.024 | 0.021 |
| rs7854982 | -0.015 | 0.003 | 0.000 | 0.003 | -0.019 | 0.018 |
| rs7945718 | 0.015 | 0.003 | -0.003 | 0.003 | 0.021 | 0.019 |
| rs7955289 | 0.017 | 0.003 | -0.006 | 0.003 | 0.027 | 0.019 |
| rs895606 | 0.015 | 0.003 | 0.002 | 0.003 | 0.075 | 0.018 |
| rs9537821 | 0.024 | 0.003 | -0.006 | 0.003 | 0.073 | 0.020 |

^a^ Educational attainment from the Social Science Genetic Association Consortium GWAS; ^b^ Number of children data from UK Biobank; ^c^ Age at first birth data from UK Biobank.

**Table S3.** Participant characteristics in UK Biobank sample for analysis.

|  | **Total N** | **Mean (SD) or n (%)** |
| --- | --- | --- |
| Sex |  |  |
| Females | 337,104 | 181,362 (53.80) |
| Males |  | 155,742 (46.20) |
| Age at assessment, years | 337,104 | 56.87 (8.00) |
| Educational attainment, years | 333,975 | 13.34 (4.44) |
| College |  |  |
| No | 333,975 | 251,951 (75.44) |
| Yes |  | 82,024 (24.56) |
| Number of children | 335,758 | 1.79 (1.20) |
| Childless |  |  |
| No | 335,758 | 270,084 (80.44) |
| Yes |  | 65,674 (19.56) |
| Age at first birth, years | 124,093 | 25.39 (4.54) |
| Number of sexual partners | 275,700 | 5.76 (8.63) |
| Highest number of sexual partners |  |  |
| No | 275,700 | 244,132 (88.55) |
| Yes |  | 31,568 (11.45) |

**Table S4. LD Score Regressions of genetic liability of schizophrenia and genetically predicted educational attainment on number of children, age at first birth and number of sexual partners using outcome summary statistics adjusted for genotype array.**

|  | **No. of offspring^a^** | | | **Age at first birth^b^** | | | **Number of sexual partners^c^** | | |
| --- | --- | --- | --- | --- | --- | --- | --- | --- | --- |
|  | **r_g_** | **se** | ***P*** | **r_g_** | **se** | ***P*** | **r_g_** | **se** | ***P*** |
| Genetic liability of schizophrenia^d^ | 0.002 | 0.008 | 0.84 | -0.007 | 0.009 | 0.44 | 0.007 | 0.009 | 0.43 |
| Genetically predicted educational attainment^e^ | -0.347 | 0.026 | 8.86x10^-41^ | 0.806 | 0.019 | <5×10^-41^ | - | - | - |

^a^ Number of children data from UK Biobank (N = 333,628); ^b^ Age at first birth data from UK Biobank (N = 123,310); Number of sexual partners data from UK Biobank (N = 273,970); ^d^ Schizophrenia data from the Psychiatric Genomics Consortium GWAS (N= 35,123 cases and 109,657 controls); ^e^ Educational attainment from the Social Science Genetic Association Consortium GWAS (N = 283,723). There were 1,114,456 SNPs included in schizophrenia analyses and 1,117,154 included in educational attainment analyses.

**Table S5. Estimates of the causal effect of genetic liability for schizophrenia and genetically predicted educational attainment on number of children, age at first birth and number of sexual partners using inverse variance weighted, mode-based estimator, MR-Egger and weighted median Mendelian randomization approaches with outcome statistics adjusted for genotype array.**

|  | **No. of offspring^b^** | **Age at first birth^c^** | **Number of sexual partners^d^** | **Childlessness^e^** | **Highest number of sexual partners^f^** |
| --- | --- | --- | --- | --- | --- |
| **Method** | **β (95% CI), *P*** | | | **OR (95% CI), *P*** | |
| **Genetic liability of schizophrenia: 101 SNPs^a^** | | | | |  |
| Inverse Variance Weighted | 0.003 (-0.003, 0.009), 0.39 | -0.004 (-0.043, 0.035), 0.84 | 0.165 (0.117, 0.212), 5.18x10^-10^ | 0.998 (0.985, 1.012), 0.79 | 1.057 (1.038, 1.077), 4.49x10^-8^ |
| MR-Egger intercept | -0.001 (-0.004, 0.001), 0.29 | -0.016 (-0.031, -0.001), 0.04 | -0.005 (-0.024, 0.013), 0.58 | 0.998 (0.993, 1.004), 0.54 | 0.994 (0.987, 1.001), 0.08 |
| Mr-egger slope | 0.020 (-0.013, 0.053), 0.23 | 0.206 (0.0001, 0.412), 0.05 | 0.235 (-0.018, 0.488). 0.06 | 1.020 (0.950, 1.094), 0.58 | 1.154 (1.045, 1.274), 0.001 |
| Weighted Median | 0.006 (-0.003, 0.015), 0.22 | 0.018 (-0.047, 0.083), 0.59 | 0.171 (0.091, 0.250), 5.54x10^-5^ | 0.996 (0.975, 1.017), 0.68 | 1.035 (1.003, 1.068), 0.04 |
| Simple MBE | 0.020 (-0.008, 0.054), 0.25 | 0.058 (-0.197, 0.312), 0.66 | 0.372 (-0.081, 0.826), 0.11 | 0.989 (0.912, 1.071), 0.78 | 1.118 (0.973, 1.284), 0.12 |
| Weighted MBE | 0.020 (-0.011, 0.052), 0.21 | 0.050 (-0.175, 0.275), 0.66 | 0.385 (-0.034. 0.805), 0.08 | 0.993 (0.924, 1.068), 0.85 | 1.011 (0.884, 1.155), 0.88 |
| **Genetically predicted educational attainment: 67 SNPs^e^** | | | | |  |
| Inverse Variance Weighted | -0.162 (-0.206, -0.118), 3.64x10^-10^ | 2.663 (2.388, 2.938), <5×10^-14^ | - | 1.590 (1.447, 1.747), 1.53x10^-14^ | - |
| MR-Egger intercept | 0.004 (0.001, 0.008), 2.50x10^-02^ | -0.031 (-0.054, -0.008), 9.48x10^-03^ | - | 0.989 (0.982, 0.997), 0.010 | - |
| Mr-egger slope | -0.391 (-0.595, -0.187), 2.99x10^-04^ | 4.348 (3.056, 5.611), 4.36x10^-09^ | - | 2.817 (1.816, 4.370), 1.35x10^-05^ | - |
| Weighted Median | -0.206 (-0.278, -0.134), 4.58x10^-07^ | 2.842 (2.378, 3.306), <5×10^-14^ | - | 1.570 (1.349, 1.828), 1.90x10^-07^ | - |
| Simple MBE | -0.253 (-0.506, 0.001), 0.06 | 3.407 (1.849, 4.966), 6.08x10^-05^ | - | 1.469 (0.884, 2.439), 0.14 | - |
| Weighted MBE | -0.249 (-0.477, -0.021), 0.04 | 1.621 (0.260, 2.981), 2.26x10^-02^ | - | 1.520 (0.964, 2.399), 0.08 | - |

^a^ Schizophrenia genetic data from the Psychiatric Genomics Consortium GWAS (N= 35,123 cases and 109,657 controls); ^b^ Number of children data from UK Biobank (N = 318,921 – 335,758 for genetic liability of schizophrenia analysis and 268,658 – 335,758 for educational attainment analysis). ^c^ Age at first birth data from UK Biobank (N = 117,844 – 124,093 for genetic liability of schizophrenia analysis and 99,317 – 124,093 for education analysis). ^d^ Number of sexual partners data from UK Biobank (N = 261,931- 275,700); ^e^ Childlessness data from UK Biobank (N = 318,921 – 335,758 for genetic liability of schizophrenia analysis and 268,658 – 335,758 for educational attainment analysis). Childlessness was coded as 1. ^f^ Highest number of sexual partners data from UK Biobank (N = 261,931- 275,700). Highest tenth percentile was coded as 1. Schizophrenia results were multiplied by 0.693 to represent the estimate per doubling in odds of the binary exposure. Results were converted to ORs for schizophrenia by multiplying log ORs by 0.693 and then exponentiating to represent the OR per doubling in odds of the binary exposure. Results were converted to ORs for educational attainment by exponentiating log ORs; ^g^ Educational attainment from the Social Science Genetic Association Consortium GWAS (N = 283,723). It should be noted that the I^2^_GX_ statistic for an unweighted MR-Egger regression was 0.33 for educational attainment and 0.20 for genetic liability of schizophrenia, which is deemed too low to conduct a SIMEX adjustment, and MR-Egger results should be treated with caution (2).

**Figure S1.** Genetic score for schizophrenia liability (in deciles) and mean number of children in UK Biobank data.


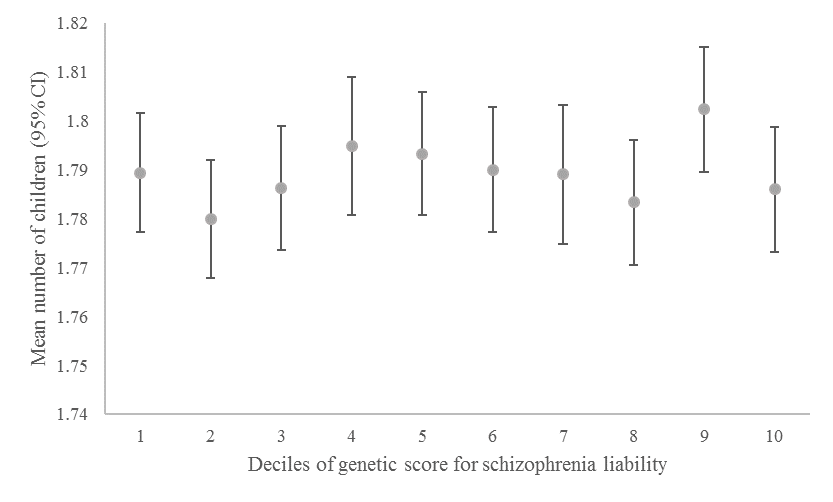


**Figure S2.** Genetic score for schizophrenia liability (in deciles) and mean age at first birth in women from UK Biobank data.

**
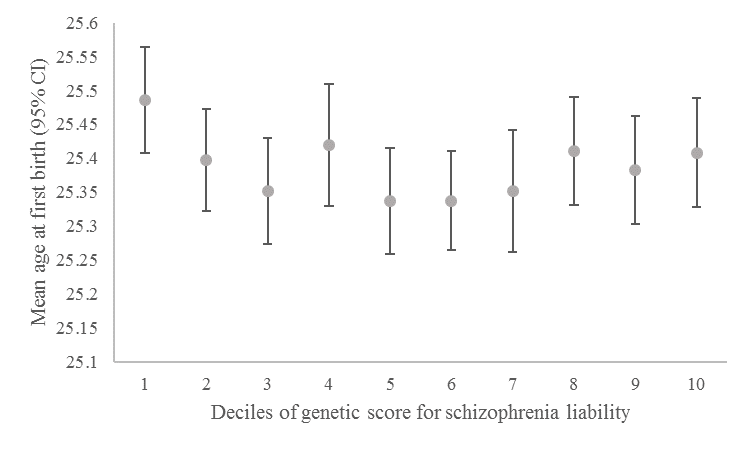
**

**Figure S3.** Genetic score for schizophrenia liability (in deciles) and mean number of sexual partners in UK Biobank data.


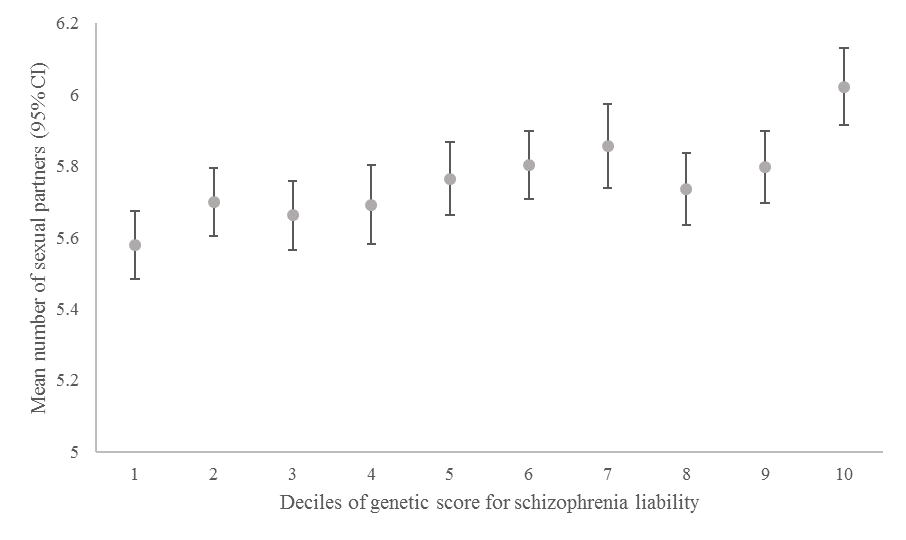


**Figure S4.** Number of children and mean genetic score for schizophrenia liability in UK Biobank data.


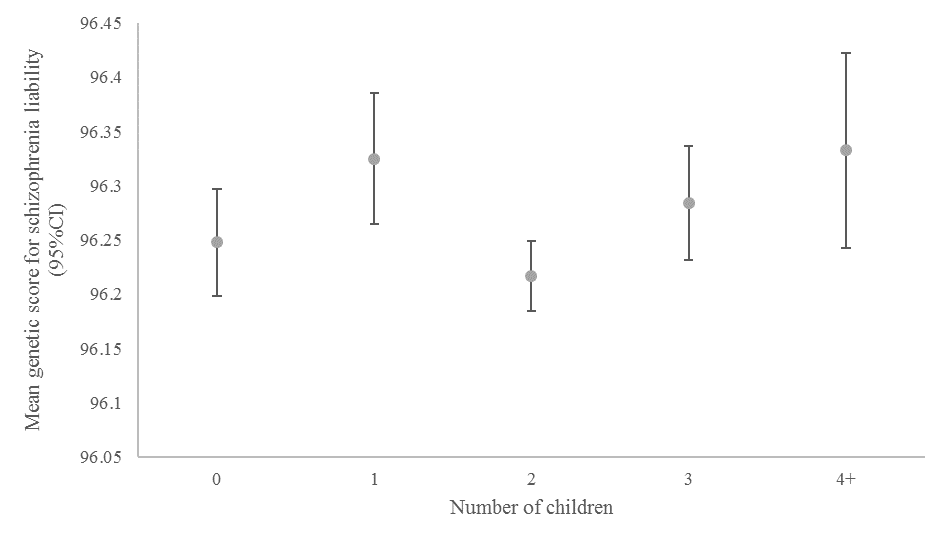


**Figure S5.** Categories of age at first birth and mean genetic score for schizophrenia liability in UK Biobank data.


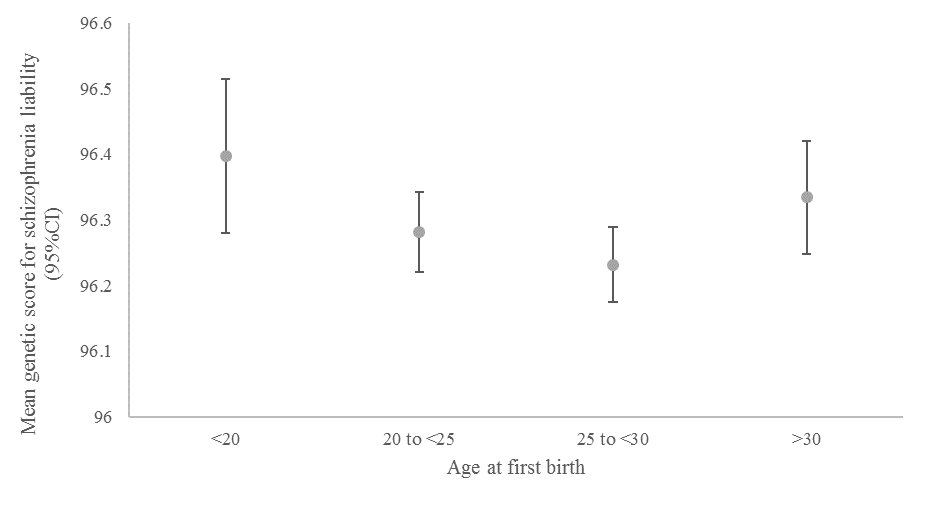


**Figure S6.** Categories for whether individuals had the highest number of sexual partners and mean genetic score for schizophrenia liability in UK Biobank data.

**
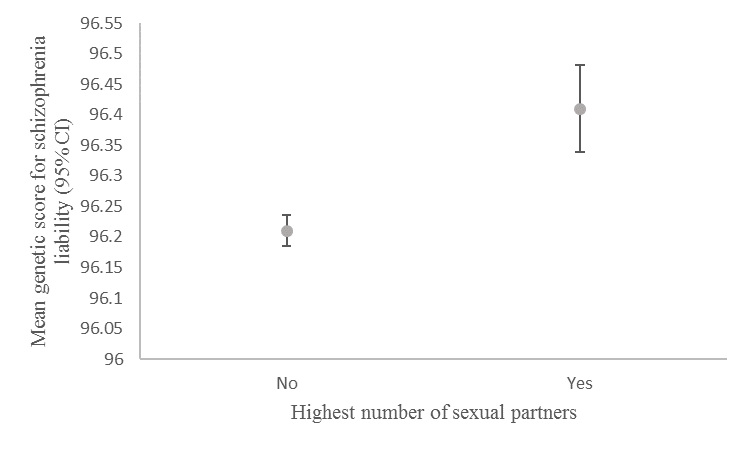
**

**Table S6.** Associations of the score for genetic liability for schizophrenia and outcomes removing cumulative deciles of the score, with cases of schizophrenia removed. Adjusted for the top 10 principal components.

|  | **Number of children** | **Age at first birth** | **Number of sexual partners** |
| --- | --- | --- | --- |
| **Schizophrenia genetic score** | **β (95% CI), *P*** | | |
| Highest 10% removed | 0.0005 (-0.0002, 0.0013), 0.18  N = 302,011 | -0.004 (-0.008, 0.001), 0.15  N = 111,599 | 0.011 (0.005, 0.017), 5.05×10^-4^  N = 248,143 |
| Highest 20% removed | 0.0002 (-0.0007, 0.0011), 0.69  N = 268,439 | -0.005 (-0.010, 0.001), 0.15  N = 99,106 | 0.012 (0.005, 0.019), 1.00×10^-3^  N = 220,745 |
| Highest 30% removed | 0.0005 (-0.0006, 0.0016), 0.37  N = 234,886 | -0.008 (-0.014, -0.001), 0.03  N = 86,593 | 0.017 (0.008, 0.025), 1.03×10^-4^  N = 193,259 |
| Highest 40% removed | 0.0006 (-0.0006, 0.0019), 0.32  N = 208,339 | -0.009 (-0.016, -0.001), 0.02  N = 76,821 | 0.015 (0.006, 0.025), 1.82×10^-3^  N = 171,511 |
| Highest 50% removed | 0.0008 (-0.0006, 0.0023), 0.29  N = 167,780 | -0.008 (-0.018, 0.001). 0.08  N = 61,811 | 0.012 (0.001, 0.024), 0.03  N = 138,230 |

**Figure S7.** Curve for prediction for fitness from a linear regression of a genetic score for schizophrenia liability on number of children and a squared genetic score for schizophrenia liability in both sexes.

**Figure S8.** Curve for prediction for fitness from a linear regression of a genetic score for schizophrenia liability on number of children and a squared genetic score for schizophrenia liability in females.

**Figure S9.** Curve for prediction for fitness from a linear regression of a genetic score for schizophrenia liability on number of children and a squared genetic score for schizophrenia liability in males.

**Figure S10.** Curve for prediction from a linear regression of a genetic score for schizophrenia liability on age at first birth and a squared genetic score for schizophrenia liability in females.

**Figure S11.** Curve for prediction from a linear regression of a genetic score for schizophrenia liability on number of sexual partners and a squared genetic score for schizophrenia liability in both sexes.

**Figure S12.** Curve for prediction from a linear regression of a genetic score for schizophrenia liability on number of sexual partners and a squared genetic score for schizophrenia liability in females.

**Figure S13.** Curve for prediction from a linear regression of a genetic score for schizophrenia liability on number of sexual partners and a squared genetic score for schizophrenia liability in males.

1. Gage SH, Jones HJ, Taylor AE, Burgess S, Zammit S, Munafò MR. Investigating causality in associations between smoking initiation and schizophrenia using Mendelian randomization. Sci Rep. 2017;7:40653.
2. Bowden J, Fabiola Del Greco M, Minelli C, Davey Smith G, Sheehan NA, Thompson JR. Assessing the suitability of summary data for two-sample mendelian randomization analyses using MR-Egger regression: The role of the I2 statistic. Int J Epidemiol. 2016;45(6):1961–74.
